# The value of left ventricular strain–volume loops in predicting response to cardiac resynchronization therapy

**DOI:** 10.1186/s12947-019-0153-3

**Published:** 2019-02-18

**Authors:** Mengruo Zhu, Haiyan Chen, Zibire Fulati, Yang Liu, Yangang Su, Xianhong Shu

**Affiliations:** 1 Department of Echocardiography, Zhongshan Hospital, Fudan University, Shanghai Institute of Cardiovascular Diseases, Shanghai Institute of Medical Imaging, 180 Fenglin Road, Shanghai, 200032 China; 2 Department of Cardiology, Zhongshan Hospital, Fudan University; Shanghai Institute of Cardiovascular Diseases, 180 Fenglin Road, Shanghai, 200032 China

**Keywords:** Cardiac resynchronization therapy, Heart failure, Strain–volume loop, Segmental heterogeneity, Wasted septal work

## Abstract

**Background:**

Three-dimensional (3D) speckle tracking imaging (STI) allows the simultaneous assessment of left ventricular (LV) strain and volume. We aim to explore the value of LV strain–volume loops in predicting response to cardiac resynchronization therapy (CRT).

**Methods:**

Forty heart failure (HF) patients scheduled for CRT and twenty healthy individuals were enrolled. All subjects received a 3D echocardiography and 3D STI analysis to acquire LV global and segmental principal strain (PS) and volume simultaneously. Values were plotted in a Cartesian system to construct PS–volume loop which was assessed using the two characteristics of the linear fitting curve: the slope and the coefficient of determination (R^2^-S/D coupling).

**Results:**

HF patients at baseline showed significantly lower slope and R^2^-S/D coupling of all PS–volume loops than healthy subjects. As for as comparing Segmental PS–Global volume loop at baseline, Midseptal R^2^-S/D coupling was lower and Midlateral slope was higher in CRT responders than in non-responders. For each individual, the abnormal segmental heterogeneity of Midseptal slope and R^2^-S/D coupling were lower than Midlateral was observed only in responders. At follow-up, significant improvements of the Midseptal slope and R^2^-S/D coupling were observed in responders. Midseptal R^2^-S/D coupling at baseline was an independent predictor of CRT response and the cut-off value of 0.55 was recommended with sensitivity of 89% and specificity of 77%.

**Conclusions:**

Analysis of strain–volume loops could provide unique information for predicting response to CRT. Assessment of septal myocardial wasted work at baseline is helpful to improve patient selection for CRT.

## Introduction

Cardiac resynchronization therapy (CRT) is an established treatment for patients with dyssynchronous heart failure (DHF) [1], meanwhile growing evidence supports that the secondary changes of CRT in molecular and cellular play an important role in reversing left ventricular (LV) remodeling. Previous study [2] has demonstrated that molecular polarization within the dyssynchronous LV was consistent with segmental heterogeneity of myocardial load distribution. Strain describes exactly myocardial deformation under the action of hemodynamic load. However, most of segmental strain analysis proposed previously [3] neglected the dynamic relationship of strain with volume load changing across the cardiac cycle. As suggested in Carasso’s study [4], LV segmental strain**–**time curve morphology is actually highly predictive of response to CRT, while the same values of strain peaks or timings could be observed with different strain curve morphologies [5].

In our study, we hypothesized that the new approach, based on the simultaneous strain–volume analysis which combining temporal changing data from function and structure, could provide unique information for predicting CRT response. Three-dimensional (3D) speckle tracking imaging (STI) allows the simultaneous evaluation of cardiac principal strain (PS) and volume changes frame-by-frame. Since 3D PS overcoming limitations of geometry-dependent reference directions (i.e. longitudinal, radial, and circumferential) [6, 7], it has been proven that 3D PS correlated well with cardiac muscle fiber arrangements and could more accurately detect regional myocardial mechanical alterations [8, 9]. To validate our hypothesis, data of each patient across one cardiac cycle were plotted in a Cartesian system to develop the PS–volume loop which was analyzed by two characteristics of the linear fitting curve: the slope and the coefficient of determination which reflecting the degree of systolic–diastolic coupling (R^2^-S/D coupling). Slope reflects dynamic relationship between strain responses alongside volume changing. As to the R^2^-S/D coupling, the smaller its value is, the severer the degree of systolic–diastolic uncoupling is, which indicating that (global or segmental) myocardial efficiency reduced in dyssynchronous LV because myocardial systolic shortening and diastolic lengthening doesn’t synchronize with chamber volume decreasing and increasing.

## Methods

### Study population

A total of 40 consecutive HF patients who were firstly scheduled for CRT were prospectively enrolled. Indications for CRT according to the 2016 European Society of Cardiology (ESC) guidelines [10] were as follow: symptomatic patients with HF with a QRS duration ≥130 ms and LVEF ≤35%, in NYHA functional class III or IV despite optimal medical treatment. Patients were excluded for the following reasons: narrow QRS, right bundle branch block (RBBB), a history of cardiac surgery, absence of clinical follow-up and poor echocardiography images. Left bundle branch block (LBBB) was diagnosed according to the criteria proposed by 2013 ESC guidelines [11] Class 1 Recommendation for CRT, namely a wide QRS duration with QS or rS in V1, broad (frequently notched or slurred) R wave in leads I, aVL, V5 or V6, and absence of q waves in leads V5 and V6. Intraventricular conduction delay [12] was diagnosed as non-specific manner QRS morphology that did not fit the criteria for LBBB and RBBB. Response to CRT was defined as a reduction in LV end-systolic volume ≥ 15% at 6-month follow-up in comparison with baseline value measured by echocardiography.

Control subjects, matched for age and gender, were selected without documented cardiovascular diseases and history of cardiovascular medication and with normal cardiac function using the American Society of Cardiology (ASE) guidelines for cardiac chamber quantification [13].

This study was approved by the medical ethics committee of our hospital, and informed consent was obtained from all subjects.

### Echocardiography

All subjects in the left lateral decubitus, with a synchronous Electrocardiogram connected, underwent transthoracic echocardiography using a Philips iE33 ultrasound machine (Philips Medical Systems, Eindhoven, The Netherlands) equipped with a S5–1 and X3–1 probe. A complete and standard 2D echocardiographic assessment and real-time 3D echocardiography were performed on HF patients before CRT device implantation and then 6-month follow-up after CRT as well as healthy controls.

### Two-dimensional echocardiography

Conventional 2D, M-mode and Doppler echocardiographic parameters were measured according to the recommendations of ASE guidelines [13].

### Three-dimensional speckle-tracking imaging

**Fig. 1:**
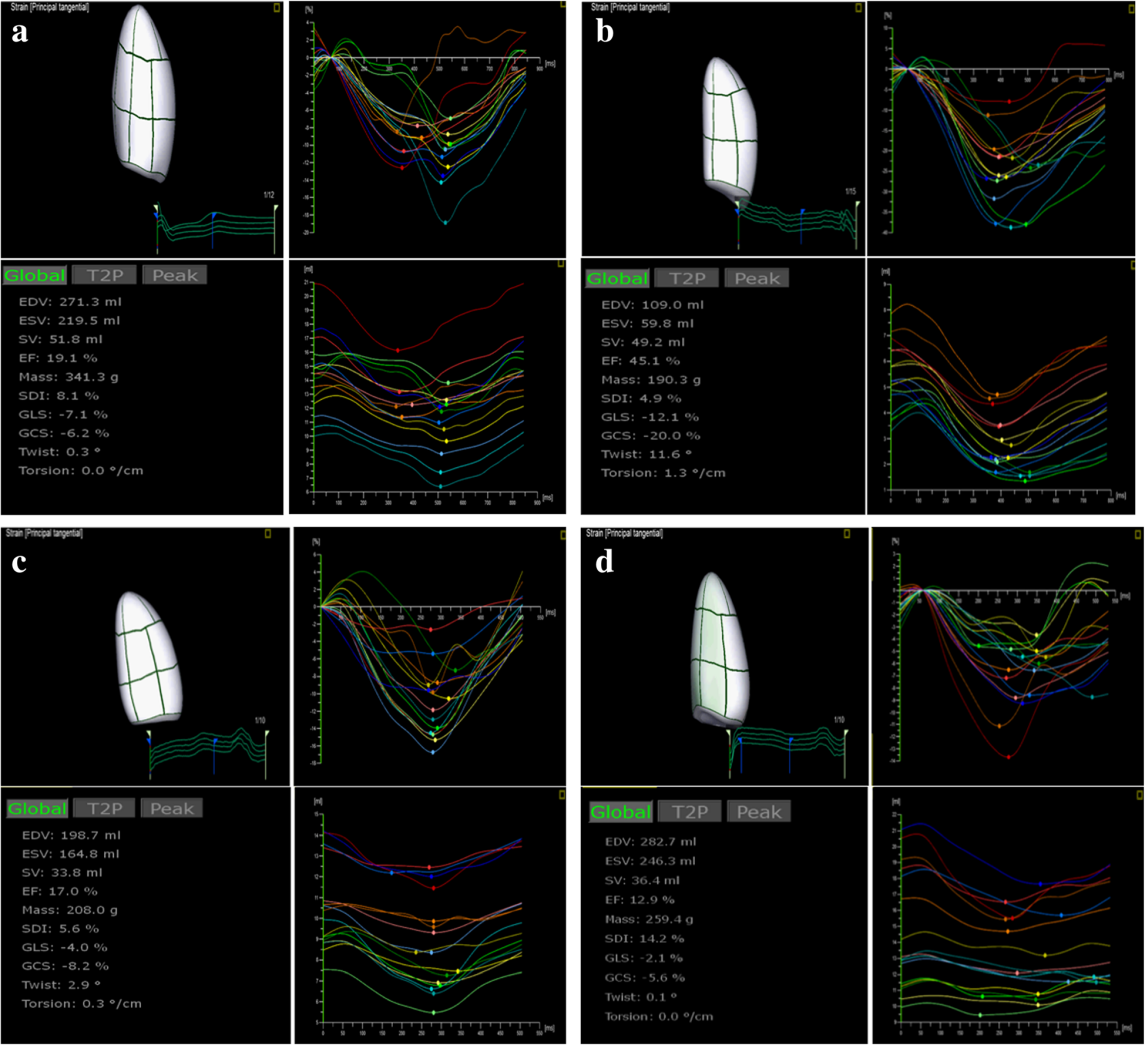
Three-dimensional speckle-tracking imaging of individual examples from a CRT responder and a non-reponder before CRT and 6-month follow-up after CRT. (**a**) A CRT responder before CRT; (**b**) A CRT responder at 6-month follow-up after CRT; (**c**) A CRT non-responder before CRT; (**d**) A CRT non-responder at 6-month follow-up after CRT. The image upper right is 16 segmental strain-time curves and lower right is 16 segmental volume-time curves

3D images were imported to the 4D speckle-tracking workstation, TomTec-Image Arena 4D Cardiac Performance Analysis; TomTec Imaging System, GMBH, Germany) and analyzed according to the following steps: First, the best cardiac cycle of the full-volume 3D acquisition was chosen, orientating one reference point to the aortic valve annulus in short-axis reference plane and two reference points to make line markers centered in LV cavity from apex to mitral valve annulus in the three apical views (four-chamber, two-chamber and long-axis) to allow the LV long axis was designated and an exact volume reconstruction. Then the software automatically distinguished the LV endocardial border and tracked it for an entire cardiac cycle. An epicardial surface tracing could be generated by the system, which was manually adjusted to cover the full thickness of the LV wall. Before processing, a cine loop preview feature visually confirmed that the internal line followed the endocardium throughout the cardiac cycle. If tracking of the LV wall was unsatisfactory, manual adjustments were made. Last, the curves of global volume, 16 segmental volume and PS were produced automatically using the standard 16-segment model (Fig. 1). Global PS was calculated by averaging 16 segmental strain components. LV end-diastolic frame time and end-systolic frame time were located as the onset of QRS wave and the end of T wave respectively according to the electrocardiogram, which identified by the software automatically.

LV end-diastolic volume (EDV), end-systolic volume (ESV), ejection fraction (EF), LV mass, and global longitudinal strain (GLS), global circumferential strain (GCS), as well as twist, torsion were automatically calculated. Strain delay index (SDI), according to the method described previously [14], was determined as the sum of the difference between peak and end-systolic PS across 16 segments. Corrected by the R-R interval, the standard deviation of time to peak PS (TpPS-SD%) and the standard deviation of time to minimum systolic volume (Tmsv-SD%) were calculated using the standard 16-segment model.

### Strain–volume loop reconstruction

**Fig. 2:**
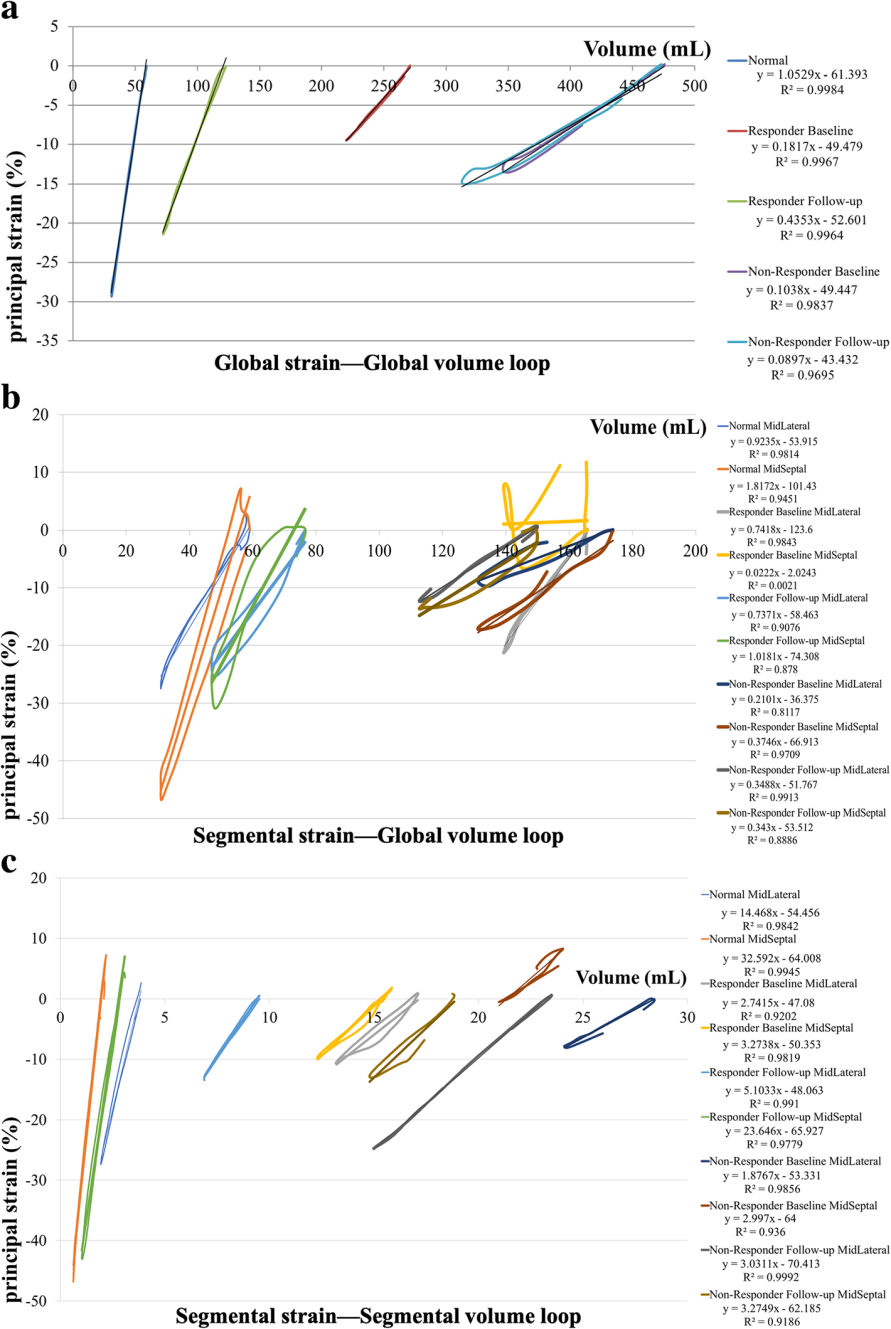
Strain–volume loops and the linear fitting curves of individual examples from a healthy subject, a CRT responder and a non-responder at baseline and follow-up. (**a**) Global strain–Global volume loop; (**b**) Segmental (Midseptal and Midlateral) strain–Global volume loop; (**c**) Midseptal strain–Midseptal volume loop and Midlateral strain–Midlateral volume loop

The raw data were exported to a spreadsheet (Excel, Microsoft Corp, Washington, US). For each individual, data were plotted as PS (y-axis) against volume (x-axis) of each frame in a Cartesian system to develop the PS–volume loop across one cardiac cycle including systolic and diastolic components which was distinguished by the end-systolic frame time determined by the electrocardiogram: Global PS–Global volume loop, Segmental (Midseptal and Midlateral) PS–Global volume loop, as well as Segmental PS–Segmental volume loop including Midseptal PS–Midseptal volume loop and Midlateral PS–Midlateral volume loop (Fig. 2).

A linear fitting line was applied to each PS–volume loop and a polynomial regression analysis of the order y = kx + c was performed on the linear fitting line. The PS–volume loop was analyzed by two characteristics of the linear fitting curve: the slope and the coefficient of determination (R^2^-S/D coupling).

### Inter- and intra-observer variability

**Fig. 3:**
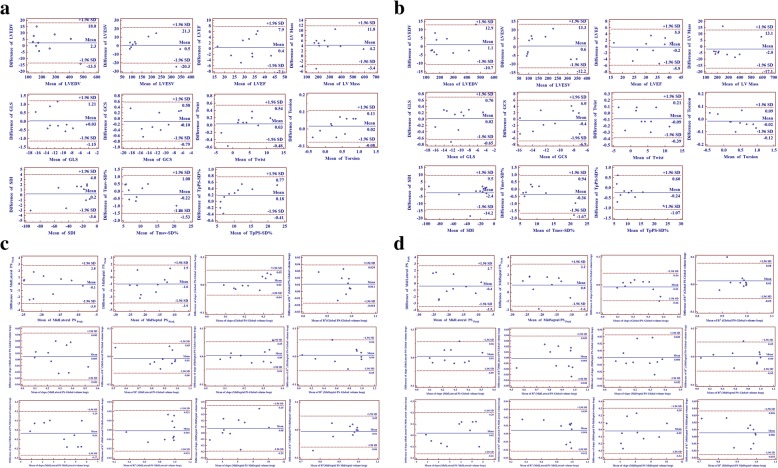
Bland-Altman analysis for inter-observer variability and inter-observer variability of all three-dimensional (3D) speckle-tracking imaging measures. (**a**) Inter-variability of conventional 3D echocardiographic parameters; (**b**) Intra-variability of conventional 3D echocardiographic parameters; (**c**) Inter-variability of segmental peak strain and characteristics of strain-volume loops; (**d**) Intra-variability of segmental peak strain and characteristics of strain-volume loops. LVEDV, left ventricular end-diastolic volume; LVEF, left ventricular ejection fraction; LVESV, left ventricular end-systolic volume; GCS, global circumferential strain; GLS, global longitudinal strain; PS, principal strain; SDI, strain delay index; Tmsv-SD%, standard deviation of time to minimum systolic volume corrected by R-R interval; TpPS-SD%, standard deviation of time to peak principal strain corrected by R-R interval

Inter- and intra-observer variability of all 3D speckle-tracking imaging measures were assessed using Bland-Altman plots (Fig. 3) with data from 10 randomly selected study 3D images, examined twice by a second observer who was blinded to the values obtained by the first observer and by one observer twice who was blinded to the results of the previous measurements, respectively.

### Statistics analysis

Continuous data are expressed as mean ± SD and dichotomous data as numbers and percentages. Dichotomized comparisons were assessed using Chi-square test or Fisher exact test as appropriate. Comparisons of values between control group and HF patients or values between responders and non-responders at baseline or 6-month follow-up were performed using the Independent-Samples T Test, while intragroup (responders or non-responders) comparisons of values between baseline state and 6-month follow-up, or characteristics of PS–volume loops between Midseptal and Midlateral were performed using the Paired -Samples T Test. To determine independent predictors of response to CRT, logistic regression analysis was performed. Significant variables selected in univariate logistic regression analysis (*P* < 0.05) were entered into the multivariate analysis. The optimal cut-off value which combine the higher value of specificity plus sensitivity was obtained through receiver operating characteristic (ROC) curve. Correlations between two variables were analyzed using Pearson’s test.

A two-sided *P* value < 0.05 was accepted as indicating statistical significance. All data were analyzed using SPSS version 24.0 (SPSS Inc., Chicago, IL, USA) and MedCalc version 12.5.0.0 (MedCalc Software, Mariakerke, Belgium).

## Results

### Study population

Twenty healthy subjects (62.9 ± 8.8 years, 30% female, 74.3 ± 9.5 beats/min) as control group and forty HF patients scheduled for CRT (60.3 ± 11.9 years, 25% female, 75.6 ± 16.3 beats/min) were included in this study. Of the 40 HF patients at 6-month follow-up, 27 patients (68%) were classified as responders while 13 patients (32%) were classified as non-responders. Baseline clinical characteristics of CRT responders versus non-responders are shown in Table 1, but no significant differences were observed in baseline clinical characteristics between them. Comparisons of 2D and 3D echocardiographic characteristics between controls and HF patients as well as between CRT responders and non-responders at baseline and at follow-up are presented in Table 2**.** Obviously, most structural parameters had been significantly changed and functional parameters had been significantly impaired in HF patients at baseline evaluation when compared with the control group. All HF patients were on stable, optimal medical treatment according to the ESC guidelines [10]. Baseline echocardiographic characteristics were comparable between responders and non-responders, except for better response among those patients with smaller LVESD and LVEDV. At 6-month follow-up, responders showed a significant decrease in LV size (diameter and volume) and LV dyssynchrony indices (TpPS-SD% and Tmsv-SD%), and a significant increase in the absolute value of GLS and GCS, whereas non-responders didn’t have any significant changes.

**Table 1:** Baseline clinical characteristics of CRT responders versus non-responders

Variable	Responders	Non-Responders	*P* Value
	(*n* = 27)	(*n* = 13)	
Age, years	61.5 ± 10	58.2 ± 15.2	.444
Female gender, *n* (%)	9 (33%)	1 (8%)	.113
Heart rate, beats/min	73.5 ± 11.5	79.6 ± 23.1	.407
SBP, mm Hg	121.8 ± 13	115.8 ± 18.6	.273
DBP, mm Hg	76 ± 8.3	70.5 ± 7.4	.065
NYHA functional class III/IV	23/4	8/5	.685
Ischemic Etiology, *n* (%)	7 (26%)	5 (38%)	.709
QRS			
duration (ms)	160.5 ± 24.1	158.3 ± 28.5	.812
LBBB morphology, *n* (%)	20 (74%)	9 (69%)	.706
Mitral regurgitation > grade II, *n* (%)	15 (56%)	9 (77%)	.463
Hypertension, *n* (%)	14 (52%)	3 (23%)	.163
Diabetes, *n* (%)	6 (22%)	2 (15%)	1.000
Renal insufficiency, *n* (%)	3 (11%)	1 (8%)	1.000
Serum biomarkers			
CK-MB, U/L	11.5 ± 4.07	10.83 ± 3.69	.646
CK-MM, U/L	53.4 ± 30.13	54 ± 31.79	.958
Cre, μmol/L	84.74 ± 41.04	88.62 ± 15.48	.756
hs CRP, mg/L	7.46 ± 15.96	3.27 ± 6.2	.513
Uric acid, μmol/L	425.44 ± 148.55	479.03 ± 168.72	.340
NT-proBNP, pg/mL	2684.83 ± 2658.35	2956.5 ± 2748.22	.786
cTnT, ng/mL	0.02 ± 0.02	0.03 ± 0.02	.101
Medication, *n* (%)			
ACEI/ARBs	15 (55%)	7 (54%)	1.000
Beta-blockers	13 (48%)	6 (46%)	1.000
Diuretics and/or spironolactone	13 (48%)	6 (46%)	1.000

*ACEI* Angiotensin converting enzyme inhibitor, *ARB* Angiotensin receptor blocker, *CK-MB* Creatine kinase MB fraction, *CK-MM* Creatine kinase MM fraction, *Cre* Creatinine, *cTnT* Cardiac troponin *T*, *DBP* Diastolic blood pressure, *hs CRP* High sensitive C-reactive protein, *LBBB* Left bundle branch block, *NT-proBNP* N-terminal of the prohormone brain natriuretic peptide, *NYHA* New York Heart Association, *SBP* Systolic blood pressure

Data are expressed as number (percentage) or mean ± SD

**Table 2:** Comparisons of 2D and 3D echocardiographic characteristics

Variable	Controls	All Patients	*P* Value	Baseline			Follow-up			Baseline versus Follow-up	
				Responders	Non-responders	*P*	Responders	Non-Responders	*P*	*P* Value	*P* Value
	(*n* = 20)	(*n* = 40)		(*n* = 27)	(*n* = 13)	Value	(*n* = 27)	(n = 13)	Value	Responders	Non-responders
2D Echocardiographic Parameters											
Aortic root, mm	30.2 ± 2.7	33.9 ± 4.2	.001	34.4 ± 4.4	32.9 ± 3.7	.329	35.1 ± 5	32.9 ± 3.5	.206	.214	1.000
LA, mm	34.2 ± 2.9	47.1 ± 10.7	.000	44.7 ± 11.6	51.9 ± 6.6	.054	41.6 ± 5.6	48.9 ± 7.2	.002	.121	.200
LVEDD, mm	46.3 ± 5.1	68.9 ± 14.4	.000	68.8 ± 7.4	69.2 ± 23.1	.942	64 ± 10.1	75.8 ± 8.6	.002	.000	.392
LVESD, mm	28.7 ± 3.0	60.2 ± 8.8	.000	58 ± 7.7	64.3 ± 9.6	.042	51.1 ± 11.5	64.8 ± 9.2	.001	.000	.759
IVS, mm	8.5 ± 1.0	9.0 ± 1.4	.184	9 ± 1.5	8.8 ± 1.4	.685	9.6 ± 1.3	8.6 ± 1.4	.042	.094	.536
LVPW, mm	9.1 ± 0.8	9.3 ± 1.3	.419	9.3 ± 1.4	9.3 ± 1.3	.880	9.5 ± 1	8.9 ± 1.2	.137	.497	.137
PASP, mm Hg	30 ± 2.8	43.1 ± 15.8	.000	43.7 ± 14.7	47.1 ± 18.5	.574	32.3 ± 5.7	37.7 ± 9.7	.050	.001	.130
DT, ms	168.8 ± 26.9	176.2 ± 52.5	.494	173.9 ± 51	160.6 ± 80.8	.619	188.3 ± 45.4	147.2 ± 44.6	.028	.355	.613
S′ wave, cm/s	11.7 ± 2.2	5.8 ± 1.9	.000	6.1 ± 1.8	5.2 ± 1.9	.201	6.2 ± 1.6	4.5 ± 1.2	.003	.335	.126
3D Echocardiographic Parameters											
LVEDV, mL	59.5 ± 12.9	226.7 ± 95.8	.000	203.5 ± 78.7	271.2 ± 112.5	.045	151.5 ± 72.9	269.3 ± 106.5	.000	.000	.898
LVESV, mL	22.2 ± 7	179.8 ± 89.4	.000	158.7 ± 77.8	220.3 ± 99.3	.051	103.8 ± 61.7	208.9 ± 84.6	.000	.000	.458
LVEF, %	62.9 ± 7.9	22.9 ± 9.6	.000	24.4 ± 10.3	20 ± 7.7	.195	34.1 ± 8.5	22.9 ± 7.7	.001	.000	.165
LV Mass, g	102.4 ± 18.8	239 ± 80.1	.000	229.1 ± 61.3	258 ± 108.3	.317	184.7 ± 61	279.3 ± 117	.003	.000	.049
GLS, %	− 22.16 ± 4.33	−7.16 ± 3.38	.000	−7.89 ± 3.45	−5.77 ± 2.86	.077	−11.09 ± 2.67	−7.61 ± 3.47	.002	.000	.088
GCS, %	−30.59 ± 6.8	−8.64 ± 4.42	.000	−9.22 ± 4.98	−7.53 ± 2.97	.291	− 13.41 ± 4.71	− 8.62 ± 3.33	.004	.000	.103
Twist, °	15.63 ± 8.1	5.48 ± 5.9	.000	5.76 ± 6.94	4.89 ± 2.91	.695	5.64 ± 5.7	2.78 ± 3.53	.123	.942	.103
Torsion, °/cm	2.16 ± 1.19	0.59 ± 0.64	.000	0.63 ± 0.76	0.51 ± 0.31	.619	0.66 ± 0.68	0.28 ± 0.32	.079	.857	.094
SDI, %	−25.52 ± 20.9	− 33.44 ± 25.53	.253	−37.33 ± 28.56	−25.99 ± 17.07	.217	−35.48 ± 32.57	−20.95 ± 21.4	.173	.809	.468
Mechanical Dyssynchrony Indices											
TpPS-SD%, %	6.42 ± 2.57	11.73 ± 6.58	.000	11.2 ± 6.22	12.73 ± 7.4	.522	8.9 ± 3.26	10.15 ± 3.83	.317	.030	.320
Tmsv-SD%, %	5.71 ± 1.3	11.74 ± 6.01	.000	11.95 ± 5.7	11.33 ± 6.82	.775	8.89 ± 3.8	10.04 ± 3.88	.405	.004	.555

*DT* Deceleration time, *IVSd* Interventricular septum diastole, *LA* Left atrial, *LVEDD* Left ventricular end-diastolic diameter, *LVEDV* Left ventricular end-diastolic volume, *LVEF* Left ventricular ejection fraction, *LVESD* Left ventricular end-systolic diameter, *LVESV* Left ventricular end-systolic volume, *LVPWd* Left ventricular posterior wall diastole, *GCS* Global circumferential strain, *GLS* Global longitudinal strain, *PASP* Pulmonary artery systolic pressure, *PS* Principal strain, *SDI* Strain delay index, *Tmsv-SD%* Standard deviation of time to minimum systolic volume corrected by R-R interval, *TpPS-SD%* Standard deviation of time to peak principal strain corrected by R-R interval

Data are expressed as number (percentage) or mean ± SD

### Peak strain and strain–volume loop

Comparisons of peak strain and characteristics of strain–volume loops between controls and HF patients as well as between CRT responders and non-responders at baseline and at follow-up are shown in Table 3**.** Comparisons of peak strain and characteristics of strain–volume loops between Midlateral and Midseptal are shown in Table 4**.**

**Table 3:** Comparisons of peak strain and characteristics of strain-volume loops

Variable	Controls (*n* = 20)	All Patients (*n* = 40)	*P* Value	Baseline			Follow-up			Baseline versus Follow-up	
				Responders (*n* = 27)	Non-responders (*n* = 13)	*P* Value	Responders (*n* = 27)	Non-Responders (*n* = 13)	*P* Value	*P* Value Responders	*P* Value Non-responders
Segmental peak strain											
MidLateral PS_Peak_	−38.6 ± 11.5	−14.7 ± 7.3	.000	−17.1 ± 7	−10.1 ± 5.7	.005	−20.8 ± 6.6	−13.2 ± 5.8	.002	.030	.239
MidSeptal PS_Peak_	−51.6 ± 10.1	−12.5 ± 8	.000	−13 ± 8.8	−11.6 ± 6.5	.635	−22 ± 11	− 12.4 ± 7.8	.011	.001	.779
Characteristics of strain-volume loops											
Global PS-Global volume loop											
slope	1.08 ± 0.35	0.26 ± 0.11	.000	0.29 ± 0.11	0.21 ± 0.1	.062	0.43 ± 0.19	0.23 ± 0.1	.002	.001	.437
R^2^-S/D coupling	0.99 ± 0.01	0.97 ± 0.05	.016	0.97 ± 0.05	0.96 ± 0.06	.441	0.99 ± 0.02	0.98 ± 0.02	.765	.055	.100
Segmental PS-Global volume loop											
MidLateral PS-Global volume loop											
slope	1.06 ± 0.4	0.32 ± 0.18	.000	0.38 ± 0.17	0.19 ± 0.12	.001	0.45 ± 0.22	0.23 ± 0.11	.003	.172	.235
R^2^-S/D coupling	0.91 ± 0.08	0.79 ± 0.27	.019	0.84 ± 0.22	0.69 ± 0.34	.180	0.85 ± 0.15	0.83 ± 0.25	.839	.920	.262
MidSeptal PS-Global volume loop											
slope	1.46 ± 0.57	0.19 ± 0.24	.000	0.2 ± 0.2	0.18 ± 0.3	.782	0.48 ± 0.34	0.21 ± 0.18	.004	.001	.717
R^2^-S/D coupling	0.93 ± 0.04	0.54 ± 0.37	.000	0.44 ± 0.39	0.73 ± 0.26	.014	0.71 ± 0.29	0.66 ± 0.34	.680	.002	.636
Segmental PS-Segmental volume loop											
MidLateral PS-MidLateral volume loop											
slope	18.66 ± 7.41	3.85 ± 1.81	.000	4.39 ± 1.78	2.82 ± 1.44	.013	6.11 ± 2.88	3.21 ± 1.52	.000	.004	.184
R^2^-S/D coupling	0.97 ± 0.04	0.93 ± 0.11	.149	0.95 ± 0.09	0.89 ± 0.15	.119	0.95 ± 0.08	0.89 ± 0.18	.194	.864	.991
MidSeptal PS-MidSeptal volume loop											
slope	28.26 ± 10.34	6.05 ± 3.1	.000	6.67 ± 3.44	4.85 ± 1.93	.099	10.8 ± 7.22	4.83 ± 2.83	.002	.005	.987
R^2^-S/D coupling	0.96 ± 0.06	0.91 ± 0.1	.052	0.89 ± 0.12	0.94 ± 0.06	.108	0.93 ± 0.13	0.81 ± 0.33	.269	.353	.215

*PS* Principal strain

Data are expressed as mean ± SD

**Table 4:** Comparison of peak strain and characteristics of strain-volume loops between MidLateral and MidSeptal

Variable	Controls (*n* = 20)			Baseline						Follow-up					
				Responders (*n* = 27)			Non-Responders (*n* = 13)			Responders (*n* = 27)			Non-Responders (*n* = 13)		
	MidLateral	MidSeptal	*P*	MidLateral	MidSeptal	*P*	MidLateral	MidSeptal	*P*	MidLateral	MidSeptal	*P*	MidLateral	MidSeptal	*P*
Segmental PS_Peak_	−38.6 ± 11.5	−51.6 ± 10.1	.000	−17.1 ± 7	−13 ± 8.8	.077	−10.1 ± 5.7	−11.6 ± 6.5	.392	−20.8 ± 6.6	−22 ± 11	.621	−13.2 ± 5.8	−12.4 ± 7.8	.701
Characteristics of strain-volume loops															
Segmental PS-Global volume loop															
slope	1.06 ± 0.4	1.46 ± 0.57	.002	0.38 ± 0.17	0.2 ± 0.2	.002	0.19 ± 0.12	0.18 ± 0.3	.887	0.45 ± 0.22	0.48 ± 0.34	.469	0.23 ± 0.11	0.21 ± 0.18	.646
R^2^-S/D coupling	0.91 ± 0.08	0.93 ± 0.04	.373	0.84 ± 0.22	0.44 ± 0.39	.000	0.69 ± 0.34	0.73 ± 0.26	.721	0.85 ± 0.15	0.71 ± 0.29	.025	0.83 ± 0.25	0.66 ± 0.34	.203
Segmental PS-Segmental volume loop															
slope	18.66 ± 7.41	28.26 ± 10.34	.000	4.39 ± 1.78	6.67 ± 3.44	.000	2.82 ± 1.44	4.85 ± 1.93	.000	6.13 ± 2.94	10.8 ± 7.22	.001	3.21 ± 1.52	4.83 ± 2.83	.040
R^2^-S/D coupling	0.97 ± 0.04	0.96 ± 0.06	.446	0.95 ± 0.09	0.89 ± 0.12	.078	0.89 ± 0.15	0.94 ± 0.06	.241	0.95 ± 0.08	0.93 ± 0.13	.512	0.89 ± 0.18	0.81 ± 0.33	.520

*PS* Principal strain

Data are expressed as mean ± SD

### Baseline evaluation

HF patients showed significantly reduced Midlateral and Midseptal peak PS than the control group (all *P* < 0.001) (Table 3). All kinds of PS–volume loops in control group were very steep and strong systolic–diastolic coupling (R^2^-S/D coupling) (Table 3). Obviously, the slope and R^2^-S/D coupling of Global PS–Global volume loop and Segmental (Midlateral and Midseptal) PS–Global volume loop in HF patients were significantly reduced when compared with that in controls (all *P* < 0.05) (Table 3). The same observations were showed in the slope of Midlateral PS–Midlateral volume loop and Midseptal PS–Midseptal volume loop in HF patients (all *P* < 0.001), but R^2^ - S/D coupling of both were similar to that in healthy subjects (all *P* > 0.05) (Table 3).

Midlateral peak PS was significantly higher in responders than in non-responders (− 17.1 ± 7 vs. -10.1 ± 5.7, *P* = 0.005), but the difference of Midseptal peak PS didn’t reach statistical significance (*P* = 0.635) (Table 3). The slope and R^2^-S/D coupling of Global PS–Global volume loop were comparable between responders and non-responders (all *P* > 0.05) (Table 3). However, responders showed significantly lower R^2^-S/D coupling of the Midseptal PS–Global volume loop (*P* = 0.014) as well as higher slope of the Midlateral PS–Global volume loop (*P* = 0.001) and higher slope of the Midlateral PS–Midlateral volume loop (*P* = 0.013) in comparison with non-responders (Table 3).

For each individual, segmental peak PS and characteristics of PS–volume loops between the septal and lateral wall were heterogeneous, which was even observed in healthy subjects with Midseptal peak PS and slope of Midseptal PS–Global volume loop were significantly higher than those of Midlateral (*P* < 0.001 and *P* = 0.002, respectively) (Table 4). Whereas in CRT responders at baseline, although difference of peak PS between Midseptal and Midlateral did not reach statistical significance, the slope and R^2^-S/D coupling of the Midseptal PS–Global volume loop were significantly lower than those of Midlateral PS–Global volume loop (*P* = 0.002 and *P* < 0.001, respectively) (Table 4). This abnormal segmental heterogeneity, contrary to the septal-lateral relationship in healthy subjects, was observed only in CRT responders but not in non-responders at baseline.

Segmental heterogeneity between Midlateral PS–Midlateral volume loop and Midseptal PS–Midseptal volume loop at baseline didn’t differ among three groups with the slope of the Midseptal PS–Midseptal volume loop was significantly higher than that of Midlateral PS–Midlateral volume loop (all *P* < 0.001) and no significant differences were observed in R^2^ - S/D coupling between them (all *P *> 0.05) (Table 4).

### Changes at follow-up after CRT

At 6-month follow-up, significant improvements in Midseptal and Midlateral peak PS, as well as in the slope of Global PS–Global volume loop, Midseptal PS–Global volume loop, Midlateral PS–Midlateral volume loop and Midseptal PS–Midseptal volume loop were observed only in CRT responders (all *P* < 0.05) but non-responders didn’t show any significant changes (Table 3). As a result, Midseptal peak PS as well as the slope of Global PS–Global volume loop, Midseptal PS–Global volume loop and Midseptal PS–Midseptal volume loop showed significantly higher values in responders than in non-responders (all *P* < 0.05) while there was no significant difference at baseline (Table 3).

Similarly, the R^2^-S/D coupling of the Midseptal PS–Global volume loop significantly improved only in responders (*P* = 0.002), so that R^2^-S/D coupling of Midseptal PS–Global volume loop in CRT responders is no longer lower than that in non-responders (baseline vs. follow-up: *P* = 0.014 vs. *P* = 0.680) (Table 3). Although segmental heterogeneity of R^2^-S/D coupling between Midseptal PS–Global volume loop and the Midlateral PS–Global volume loop was still existed in CRT responders, it attenuated to a great extent (baseline vs. follow-up: *P* < 0.001 vs. *P* = 0.025) (Table 4). Besides, responders no longer showed abnormal segmental heterogeneity of slope between Midseptal PS–Global volume loop and Midlateral PS–Global volume loop at follow-up (baseline vs. follow-up: *P* = 0.002 vs. *P* = 0.469) (Table 4).

### Analyses to identify baseline predictors of response to CRT

**Fig. 4:**
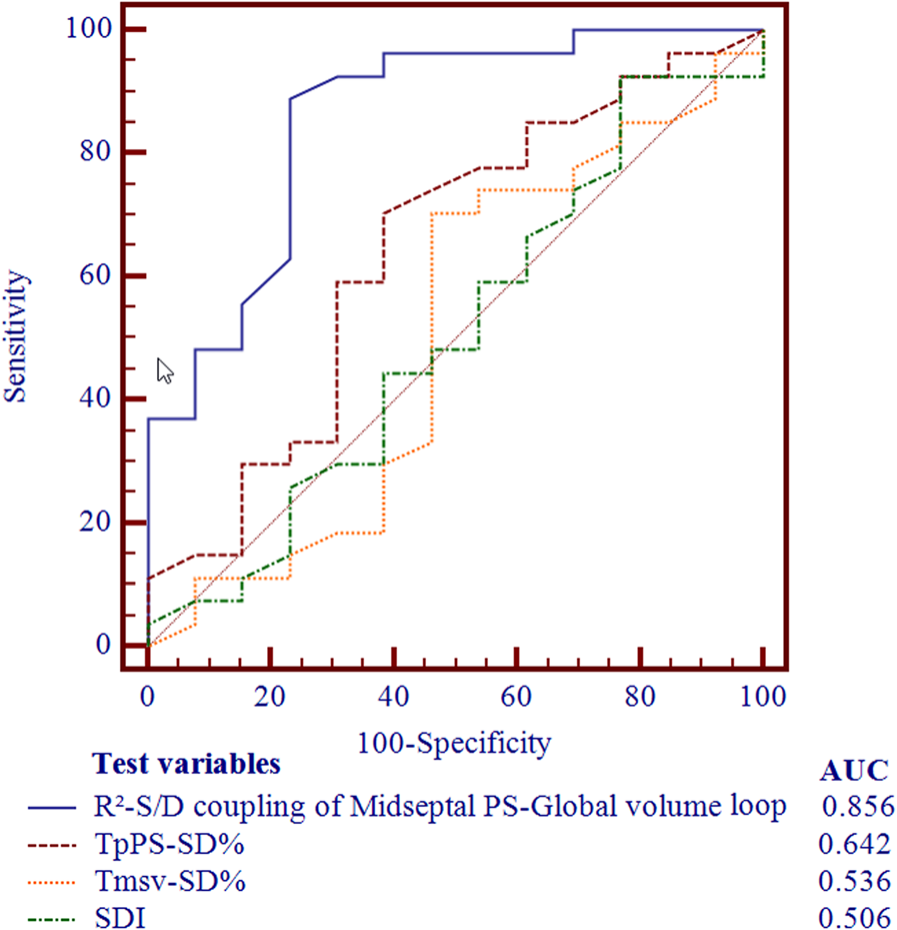
Receiver operating characteristic (ROC) curves for predicting response to cardiac resynchronization therapy (CRT). The area under the ROC curve (AUC) of R^2^-S/D coupling of Midseptal PS–Global volume loop was greater than that of TpPS-SD%, Tmsv-SD% and SDI (*P* < 0.05). SDI, strain delay index; Tmsv-SD%, standard deviation of time to minimum systolic volume corrected by R-R interval; TpPS-SD%, standard deviation of time to peak principal strain corrected by R-R interval

**Fig. 5:**
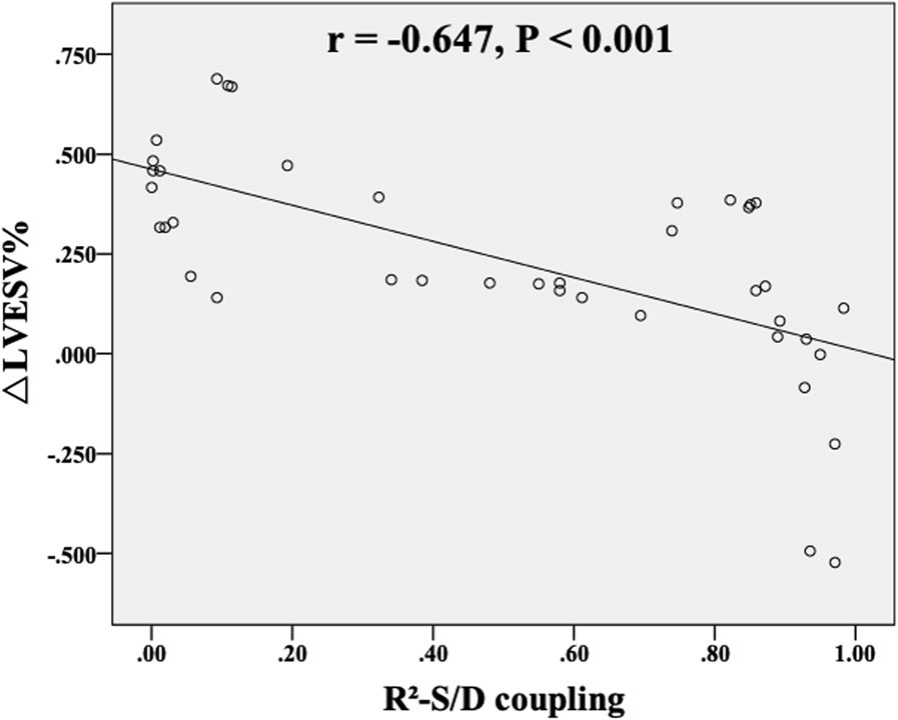
Correlation between percentage change in LVESV (△LVESV%) at 6-month follow-up and baseline R^2^-S/D coupling of Midseptal PS–Global volume loop

Logistic regression analysis was performed to identify independent predictors of response to CRT **(**Table 5**)**. All variables significantly associated with CRT response in univariate regression analysis were involved in multivariate regression analysis. R^2^-S/D coupling of Midseptal PS–Global volume loop at baseline (odds ratio 0.878, 95% CI 0.810–0.930, *P* = 0.028) was found to be an independent predictor of CRT response. The area under the ROC curve (AUC) of R^2^-S/D coupling of Midseptal PS–Global volume loop was greater than that of TpPS-SD%, Tmsv-SD% and SDI (all *P* < 0.05) **(**Fig. 4**)**. The optimal cut-off value of R^2^-S/D coupling was recommended as 0.55 (AUC value, 0.856; sensitivity, 89%; specificity, 77%) which determined by ROC curve. Besides, R^2^-S/D coupling of Midseptal PS–Global volume loop at baseline was significant correlated with percentage change in LVESV (△LVESV%) at 6-month follow-up in comparison with baseline value (*r* = − 0.647, *P* < 0.001) **(**Fig. 5**)**.

**Table 5:** Analyses to identify baseline predictors of response to CRT

Variable	Univariate Analysis			Multivariate Analysis		
	OR	95% CI	*P*	OR	95% CI	*P*
Age	1.011	1.000–1.023	.054			
Female	7.071	0.774–64.575	.083			
QRS duration	1.004	0.976–1.032	.806			
LBBB	1.417	0.310–6.470	.653			
NYHA class III	1.800	0.380–8.535	.459			
Nonischemic Etiology	1.633	0.383–6.968	.508			
NT-proBNP	1.006	0.990–1.020	.340			
hs CRP	1.001	1.000–1.070	.420			
LVESD	1.009	0.998–1.020	.123			
LVEDV	1.001	0.999–1.004	.330			
LVEF	1.003	0.930–1.082	.939			
GLS	0.892	0.809–0.984	.022	0.820	0.490–0.995	.190
GCS	0.922	0.852–0.997	.042	0.962	0.657–1.000	.660
TpPS-SD%	1.006	0.999–1.013	.112			
Tmsv-SD%	1.007	1.002–1.015	.048	1.002	1.000–1.001	.120
SDI	0.978	0.958–0.999	.041	0.990	0.680–1.000	.240
MidLateral PS_Peak_	0.818	0.697–0.960	.014	0.890	0.550–0.980	.150
Slope of MidLateral PS- Global volume loop	3.934	2.863–6.444	.016	2.880	1.230–6.700	.470
R^2^-S/D coupling of MidSeptal PS-Global volume loop	0.803	0.760–0.880	.009	0.878	0.810–0.930	.028
Slope of MidLateral PS-MidLateral volume loop	2.035	1.104–3.750	.023	1.796	0.980–3.000	.200

*CI* Confidence interval, *GCS* Global circumferential strain, *GLS* Global longitudinal strain, *hs CRP* High sensitive C-reactive protein, *LVEDV* Left ventricular end-diastolic volume, *LVESD* Left ventricular end-systolic diameter, *NT-proBNP* N-terminal of the prohormone brain natriuretic peptide, *OR* Odd ratio, *PS* Principal strain, *SDI* Strain delay index, *Tmsv-SD%* Standard deviation of time to minimum systolic volume corrected by R-R interval, *TpPS-SD%* Standard deviation of time to peak principal strain corrected by R-R interval

## Discussion

The aim of our study was to explore whether the LV strain–volume loops, a novel approach linking the structural changes to functional alterations, could provide a new perspective for assessing LV remodeling in HF patients and reverse remodeling following CRT, in the hope of offering additional information for predicting response to CRT and exploiting additive value of this approach in selection of CRT candidates.

It has been demonstrated in our study that the absolute value of GLS, GCS increased and LV mechanical dyssynchrony indices (TpPS-SD% and Tmsv-SD%) reduced after CRT. However, such echocardiographic parameters at baseline didn’t show no significant differences between responders and non-responders. Similar results were found in previous studies [15]. Methods of dyssynchrony index assessment were studied extensively in the past but with incomplete success to predict outcome. Early enthusiasm towards such methods [16, 17] was dampened by the multicenter Predictors of Response to Cardiac Resynchronization Therapy (PROSPECT) trial [18] that manifested the dyssynchrony parameters had marked variability in prediction of the clinical composite score response (sensitivity 6–74%, specificity 35–91%) and prediction of LV end-systolic volume response (≥15% reduction; sensitivity 9–77%, specificity 31–93%). SDI, which was a concept of wasted work introduced by Lim et al. [14] and has been proven to have a strong predictive value for predicting response to CRT [19], was comparable between responders and non-responders at baseline and even had no significant change after CRT in our study. All these parameters have limiting ability to predict CRT response probably because none of them incorporates dynamic changing myocardial loading and stress state. As shown in our study, Midlateral peak PS was higher in responders than in non-responders, but Midseptal peak PS didn’t differ at baseline so that we couldn’t judge whether septal was working effective or not. Besides, in view of the difference of peak PS between Midseptal and Midlateral did not reach statistical significance, we couldn’t infer that the heterogeneity of segmental strain distribution in dyssynchrony LV. Therefore, Russell et al. [20] proposed the LV pressure-strain loop analysis which allows the estimation of regional and global LV work (quantified by strain rate multiplying by instantaneous LV-pressure) and furthermore introduced the wasted work ratio as an index to predict response to CRT [21]. Vecera et al. [22] also demonstrated the wasted work in the septum calculated by similar methods was a strong predictor of response to CRT. However, clinical use of the LV pressure-strain analysis is limited by the challenge to noninvasive acquire instantaneous LV pressure.

The new method based on simultaneous strain–load (volume or area) analysis has been applied in certain physiological state [23, 24] and pathological LV remodeling [25, 26]. Besides, it has been recently validated the same method with tagging cardiac magnetic resonance [27]. It took about 3–5 min to perform strain–volume loop analysis off-line for each patient after practice in our study. So, it would be more generally applicable in clinical use by virtue of its noninvasive and convenient. Considering LV mapping data [28] have shown that the mid-septal region was electrically activated first and U-shaped conduction pattern through the apical regions was imposed on the LV activation sequence by a transmural functional line of block located between the LV septum and the lateral wall with a prolonged activation time, our study focused on septal and lateral strain–volume relationship analysis at the mid-ventricular level, which not only enhancing reproducibility since segmental heterogeneity was most evident between septum and lateral wall but also enhancing operability by simplifying evaluation and reducing data loss. Firstly, our study explored characteristics of the LV PS–volume loops in healthy subjects to verify the reliability of this new method and provide reference foundation to the establishment of evaluation criteria of LV remodeling such as in HF. As expected, the healthy subjects showed the normal myocardial shortening and lengthening was well coupled to the simultaneous progressive reduction and expansion of volume. In healthy subjects, all kinds of PS–volume loops showed steep slope, indicating a good systolic and diastolic performance. Besides, a similar strain value could be observed for any given LV volume during systole and diastole in healthy individuals, which suggesting the presence of strong systolic–diastolic coupling. This result was concordant with previous study [23].

At baseline evaluation of HF patients, all kinds of PS–volume loops showed a lower slope when compared with that in healthy subjects. As a result of the simultaneous presence of chamber dilatation (rightward shift of the loop) and strain reduction (upward shift of the loop), the two parameters were moving in the opposite direction from the normal loops in the Cartesian system and we could observe significant alteration of the slope. Furthermore, dissociation occurred between systolic and diastolic strain at the same volume, which indicating the presence of uncoupling in the relationship between strain and volume in HF patients. In dyssynchronous LV, systolic–diastolic uncoupling of strain–volume loop reflects myocardial efficiency reducing because myocardial systolic shortening and diastolic lengthening doesn’t synchronize with chamber volume decreasing and increasing, which resulting in much wasted work globally or segmentally. In addition, strain–volume loop provides an intuitive visual representation to detect the response to CRT. As shown in our study, if CRT effective, a simultaneous volume reducing (leftward shift of the loop) and PS increasing (downward shift of the loop) would make the loop steeper and better systolic–diastolic coupling.

At baseline, the R^2^-S/D coupling of the Midseptal PS–Global volume loop in CRT responders was significantly lower than that in non-responders, besides, the R^2^-S/D coupling of the Midseptal PS–Global volume loop at baseline was found to be an independent predictor of CRT response in multivariate analysis whereas dyssynchrony parameters were not. This result indicated that much wasted myocardial work was located to the septum at baseline in CRT responders. The amount of septal wasted myocardial work at baseline was related to the magnitude of benefit following CRT. The more the septal wasted at baseline, the higher probability of response to CRT was achieved. Nevertheless, the slope of the Midseptal PS–Global volume loop didn’t show significant difference between CRT responders and non-responders. While the synchronism of global and segmental myocardium maintains higher level with good systolic-diastolic coupling, myocardial contractility could be reflected by the slope of strain-volume loop. But if higher degree dyssynchrony of global and segmental myocardium is presented with systolic-diastolic uncoupling, the slope of strain-volume loop would underestimate the myocardial contractility. After CRT, the slope and R^2^-S/D coupling of the Midseptal PS–Global volume loop as well as Midseptal peak PS were increased significantly, accompanied by a significant increase in the slope of Global PS–Global volume loop as well as GLS and GRS, which suggesting an improvement in septal efficiency following CRT could contribute to the improvement in LV global function.

Midlateral peak PS as well as the slope of Midlateral PS–Global volume loop and Midlateral PS–Midlateral volume loop in CRT responders were higher than in non-responders at baseline. Therefore, we speculate that the compensatory maintenance of LV lateral wall function at a certain level was associated with a favorable response to CRT, which might be inconsistent with the view of Zweerink [29]. In Zweerink’s study, absolute values of lateral wall strain, strain rate, and work were significantly higher in responders, but this finding is not getting the attention it deserved for further investigation and deeper analysis.

In addition, our results also indicated that applying CRT to patients with abnormal segmental heterogeneity between septum and LV lateral wall, which contrary to that in healthy subjects, may be more likely to response to CRT. This heterogeneous distribution of myocardial work can be rebalanced by restoring normal electrical activation following CRT. It is helpful to improve patient selection for CRT to identify this abnormal but reversible segmental heterogeneity. Electric activation delay generates contractile dyssynchrony, with early-activated septum earlier onset of shortening during isovolumetric contraction phase against a low afterload while late-activated lateral wall shortening against increased wall stress and loading at late systole into early relaxation [30]. The early-developed septal force is dissipated in generating sufficient energy to open the aortic valve and in stretching the late-activated lateral wall. The latter event represents wasted energy during early ejection. Passive stretch might influence regional myocardial contractility because changes of effective preload triggered local Frank-Starling mechanism. The passive pre-stretch would enable the late-activated segments to contract to a greater extent in order to compensate for the increased loading conditions. Thus, septal work efficiency being reduced and lateral function compensatory being improved together generate the abnormal segmental heterogeneity. CRT could improve septal work efficiency and normalize lateral function in responders, which making heterogeneous distribution of myocardial work significantly decrease significantly or even eliminate. As observed in our study, responders showed improvements in the slope and R^2^-S/D coupling of the Midseptal PS–Global volume loop after CRT, whereas no significant change was observed in characteristics of the Midlateral PS–Global volume loop.

Most probably as the result of an optimized local loading state by CRT, the slope of both the Midseptal PS–Midseptal volume loop and the Midlateral PS–Midlateral volume loop were significantly increased in responders after CRT.

### Limitations

This study was performed in a single center, with a relatively small sample size. Therefore, we cannot draw definitive conclusions but only formulate a hypothesis that needs to be confirmed by future, larger, multi-center prospective trials.

## Conclusions

Analysis of strain–volume loops could provide unique information for predicting response to CRT and may become a potential new tool to detect the beneficial effects of CRT on LV function. Our study demonstrated that assessment of wasted work in septum at baseline would be helpful to improve patient effective selection for CRT. R^2^-S/D coupling of Midseptal PS–Global volume loop at baseline is proved to have predictive value for predicting response to CRT.

## Abbreviations

2D: two-dimensional
3D: three-dimensional
AUC: area under the curve
CI: confidence interval
CRT: cardiac resynchronization therapy
EDV: end-diastolic volume
EF: ejection fraction
ESV: end-systolic volume
HF: heart failure
LV: left ventricular
PS: principal strain
ROC: receiver operating characteristic
SDI: strain delay index
STI: speckle tracking imaging
Tmsv-SD%: standard deviation of time to minimum systolic volume corrected by R-R interval
TpPS-SD%: standard deviation of time to peak principal strain corrected by R-R interval
